# Development of Joint Activity Angle Measurement and Cloud Data Storage System

**DOI:** 10.3390/s22134684

**Published:** 2022-06-21

**Authors:** Chiu-Ching Tuan, Yi-Chao Wu, Wen-Ling Yeh, Chun-Chieh Wang, Chi-Heng Lu, Shao-Wei Wang, Jack Yang, Tsair-Fwu Lee, Hsuan-Kai Kao

**Affiliations:** 1 Department of Electronic Engineering, National Taipei University of Technology, Taipei 10608, Taiwan; cctuan@ntut.edu.tw (C.-C.T.); wwang4603@gmail.com (S.-W.W.); 2 Interdisciplinary Program of Green and Information Technology, National Taitung University, Taitung 95092, Taiwan; alanwu@nttu.edu.tw; 3 Department of Orthopedics, Lotung Poh-Ai Hospital, Yilan 26546, Taiwan; yeh610128@gmail.com; 4 Department of Radiation Oncology, Chang Gung Memorial Hospital at Linkou, Taoyuan 33305, Taiwan; jjwang@cgmh.org.tw (C.-C.W.); chiheng@cgmh.org.tw (C.-H.L.); 5 Department of Radiation Oncology, RWJ Medical School, Monmouth Medical Center, Long Branch, NJ 07740, USA; jack.yang@rwjbh.org; 6 Department of Electronics Engineering, National Kaohsiung University of Science and Technology, Kaohsiung 80708, Taiwan; tflee@nkust.edu.tw; 7 Bone and Joint Research Center, Chang Gung Memorial Hospital, Linkou 33305, Taiwan; 8 College of Medicine, Chang Gung University, Taoyuan 33305, Taiwan; 9 Department of Orthopedic Surgery, Chang Gung Memorial Hospital at Linkou, Taoyuan 33305, Taiwan

**Keywords:** elbow range of motion (ROM), extremities rehabilitation, goniometer, weighted moving average filter (WMAF), Kalman filter, cloud database

## Abstract

In this study, we developed a range of motion sensing system (ROMSS) to simulate the function of the elbow joint, with errors less than 0.76 degrees and 0.87 degrees in static and dynamic verification by the swinging and angle recognition modules, respectively. In the simulation process, the ɣ correlation coefficient of the Pearson difference between the ROMSS and the universal goniometer was 0.90, the standard deviations of the general goniometer measurements were between ±2 degrees and ±2.6 degrees, and the standard deviations between the ROMSS measurements were between ±0.5 degrees and ±1.6 degrees. With the ROMSS, a cloud database was also established; the data measured by the sensor could be uploaded to the cloud database in real-time to provide timely patient information for healthcare professionals. We also developed a mobile app for smartphones to enable patients and healthcare providers to easily trace the data in real-time. Historical data sets with joint activity angles could be retrieved to observe the progress or effectiveness of disease recovery so the quality of care could be properly assessed and maintained.

## 1. Introduction

Elbow and wrist chronic diseases often occur in musculoskeletal disorders. Aches—as a result of these chronic diseases—often affect muscle functioning and lead to reduced range(s) of motion (ROM). Since the ROM variance of the elbow joint may be slight, the uninjured side of the elbow could be referenced for an elbow injury. Moreover, the activities of elbow joints are affected by age, sex, and BMI [1,2]. By comparing the healthy side to the injured side of an elbow after six months, the average restorations of the muscle flexion and stretch were 81.25% and 92%, respectively; this shows that malfunctioning of the ROM and decreased muscle strength are often complications after a fracture [3].

A goniometer is often used to evaluate the range of angle motion in the medical field, especially for physiotherapy evaluations. However, a goniometer is constrained to evaluating the range of angle motion due to hand instability and errors from both hands of a doctor. The maximal standard error of the average total range of motion (ROM) was shown to be 11.5 degrees (regarding the reliability and validity of a universal goniometer for the adult elbow) [4]. An electronic goniometer could be used to measure the ROM of an elbow in physiotherapy and clinical evaluations. Since the ROM evaluation is more accurate and reliable with an electronic goniometer, an electronic goniometer is often used for research in laboratories [5]. However, the electronic goniometer is more expensive and complex during treatments.

The ROM evaluation in a mechanical goniometer is not accurate without dynamic measurements. An inertial motion sensor for monitoring hand motion was proposed [6], showing that the inertial motion sensor could be combined with magnetic, angular rate, and gravity sensors in a glove to objectively evaluate the hand function [6]. In [7], the authors showed that the minimal detectable difference, MMD, was smaller by a 3D motion capturing system due to lower measurement errors after evaluating the repeatability of the ROM measurements in hand joints by comparing the manual goniometer with the 3D motion capturing system. Hence, more accurate measurement methods could reduce the number of subjects for statistical significance. The evaluating values for joints (by a goniometer) should be interpreted carefully due to low repeatability and reliability [7]. Most motion radian measurements via photography are evaluated manually. The accuracies of motion radian measurement are often evaluated by observers. Hence, a ROM measurement in the elbow joint via DIPT was proposed to evaluate the validity and reliability compared with the standard ROM measurement in the elbow joint. It showed that the difference between a ROM measurement in the elbow joint by DIPT and the standard ROM measurement in the elbow joint was more than 15.99 degrees [8].

Since more apps are being designed for smart mobile phones, many clinicians are measuring ROM (i.e., elbow motion) via apps. In [9,10,11], the authors showed that ROM measurements in the elbow joint had limits of agreement, LOA, in both mobile phone inclinometers and gold standard UGs. The mean difference was from 9.4 to 12.2 degrees. In [12,13], the authors showed that the reliability and validity of ROM measurements via mobile phone cameras and manual goniometry, by surgeons, were the same in the statistical analysis. In [14], the authors showed that apps were not related to predefined buckling and supination compared with goniometry for ROM measurements. The ROM measurements by X-ray ranges estimating with surgeons were also not accurate.

Due to the progress of sensor techniques—the inertial sensor has become another measurement tool used for ROM due to its digital and precise abilities and low cost. In [15], the experimental results showed that the ROM measurements in both the elbow and wrist—by the inertial sensor—were more reliable and valid than *that* by a general goniometer. However, the reliability of a ROM measurement in the external rotation of an elbow joint was much lower due to skin artifacts. The validity of active flexion and extension ROM in the elbow by the GYKO inertial system was the same as *that* by the gold standard UG for healthy subjects [16]. In [17], the wearable woven sleeves were encapsulated with an accelerometer based on an e-textile to measure the bending angle in both the arm and knee joints. Since the electronic and optical fiber goniometer was better than the mechanical goniometer, a reliable and non-invasive PSC-ARE encoder was proposed to capture the code image by the optical mouse sensor (with low cost and high accuracy) [18]. In [19], a fiber Bragg grating goniometer, FBGG, was proposed to evaluate the articular angular motion by transforming the joint rotational motion into a cantilever strain change. The fiber Bragg grating goniometer became an efficient method to evaluate the angular motion in any kind of articular of the body.

The ROM of a joint is an important clinical parameter to evaluate the functional incapacitation of joint bleeding in hemophilia. ROM of the joint is also a remote medical tool to reduce patient reliance on hospitals. Two physical therapy plans used to evaluate manual therapy—home exercises combined with educational courses—show that the two physical therapy plans could alleviate the elbow pain for hemophilia [20]. In [21], the ROM of the elbow joint was evaluated efficiently via a Microsoft Kinect v2 sensor with angle measurement calibration (for a contactless ROM measurement). In [22], researchers used a GoNet v2-combined computer with a Kinect sensor for the ROM measurement. The results showed that the abduction of the shoulder, buckling of the shoulder and elbow, and stretching of the shoulder and elbow could all be measured accurately. In [23], a biomechanics protocol was proposed to evaluate the motion of a healthy person and patient, respectively, in daily life. The experimental results showed that Kinect v2 could be used to evaluate the patient’s motion. To ensure patient improvements (from their injuries, after several months), a clinical follow-up is required. In [24], the clinical examination of a supracondylar humeral fracture using a goniometer and hydraulic dynamometer, respectively, for a child, was proposed. Unfortunately, the experimental results in the goniometer were different from *those* in the hydraulic dynamometer (by comparing with the gold standard goniometer). The mean difference in elbow extension was 0.97 degrees. The mean difference in elbow buckling was 7.97 degrees. Remote rehabilitation has been used by some occupational therapists. The performance of the upper elbow (when comparing remote rehabilitation and traditional rehabilitation) was evaluated for elbow fracture improvement [25]. Results showed that the satisfaction reported with remote rehabilitation was much higher. Moreover, remote rehabilitation could be executed with fewer family members. The participation and compliance of measurements at home could improve for patients before and after surgery for stiff elbows (with closely monitored ROM). During recovery, with closely monitored ROM, the participation and compliance of measurements could also improve at home. In [26], the authors evaluated ROM at home by photography, movies, and apps, respectively, compared with the UG. The experimental results showed that apps in smartphones were not suitable for ROM measurements at home.

The range of motion (ROM) in a joint is an important metric to observe the status of the joint function after postoperative recovery. In the existing methods used to detect the degree of joint damage, the universal goniometer (UG) is often used to examine the degree of joint damage. However, the measurement error may be over 10 degrees due to improper positioning (caused by improper operation from the medical personnel or patient movement). Although the degree of joint damage measured by an electronic goniometer is shown to be more precise than *that* measured by a universal goniometer, an image is needed—to be recognized by both—in the follow-up image processing and evaluation (in non-real-time). Moreover, the electronic goniometer (EG) is expensive without dynamic measurement capabilities. Hence, both the UG and EG were not suitable for measuring the degree of joint damage. Although the degree of joint damage may be measured via a mobile phone, the mobile phone is difficult to be fixed to the arm for dynamic measurements. Action gloves and electronic textiles with inertial sensors could address the above issues. However, action gloves and electronic textiles were not applied for residential rehabilitation since the action glove techniques and electronic textiles were not yet widespread.

In this paper, we aimed to develop a joint activity angle measurement system combined with cloud data storage. The joint activity angle was measured accurately by sensing and recognition module sensors on the simulated arm and lower limb. A cloud database system was built for data storage from sensors via a wireless network in real-time. Thus, the medical staff could obtain the sensing data immediately. In addition, an app was designed for patients and medical staff to inquire about the data of the joint activity angle, at any time, and to track the status of the diseased limb (regarding rehabilitation). The clinical medical quality of orthopedics could be improved.

A wearable, accurate, and real-time measurement tool is the main consideration for orthopedic staff. The time of the return visit for the patient was often more than two weeks to one month after surgery. During that period of time, the medical staff could not track the recovery status or the efficacy of the rehabilitation (some patients’ conditions may thus grow worse, such as stiff limbs). The medical quality of rehabilitation also descended. Therefore, a wearable, accurate, and real-time measurement tool is proposed for patients and medical staff in this paper.

## 2. Materials and Methods

**Figure 1:**
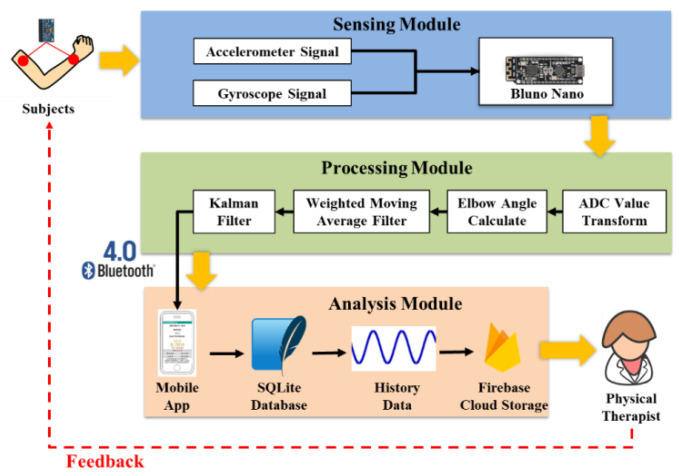
ROMSS architecture.

The 6-axis range of motion sensing system (ROMSS) was divided into the sensing module, recognition module, and feedback module, respectively. The sensing module included swing signal capturing and filtering to measure the swing angle in the upper arm. The swing signal by filtering and calibrating could be denoted by the waveform and angle of the upper arm. The waveform, high peak value, and low peak value were displayed in a GUI. The subjects could track the activity of the upper arm and joint with a figure or a table, simultaneously, from the above GUI. At the same time, the data could be uploaded to the cloud storage server, as shown in Figure 1.

**Figure 2:**
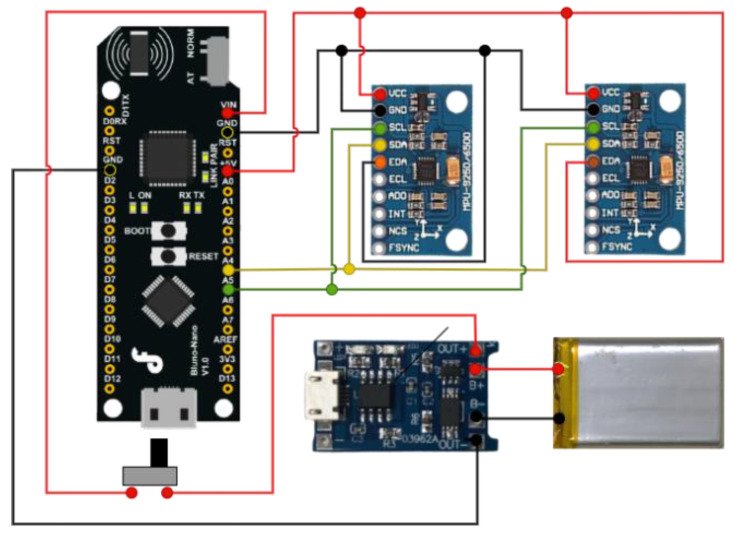
ROMSS hardware architecture.

The angle sensing system is shown in Figure 2. The angle of the elbow joint could be detected by a gyro sensor and accelerometer in the inertial sensor. The microcontroller was a Bluno Nano from DFRobot (DFRobot, Shanghai, China). The kernel of the microcontroller was ATmega328 with Bluetooth (TI CC2540). The indoor transmission range of TI CC2540 was 20 m. The signal interference could be reduced in TI CC2540. The inertial sensor was MPU 9250 from InvenSense (Invensense, San Jose, CA, USA). The inertial sensor included a 3-axis gyro sensor, a 3-axis accelerometer, and a 3-axis magnetometer. Each was equipped with a three 16-bit ADC and 400 kHz I^2^C; a 1 MHz SPI. SCL pin and an SDA pin responded to data transmission. Two different inertial sensors were distinguished by the AD0 pin.

The correct location and direction were obtained by a filter and analysis from six degrees of freedom data from the swing in free space. To obtain the optimal degrees of freedom, the carrier displacement and rotation calculation by acceleration and angular velocity were needed. The calculation could be divided into an inclination angle calculation of the 3-axis accelerometer, rotation angle calculation of the gyro sensor, Kalman filter, and filter signal identification, respectively.

After obtaining the acceleration transformed from the out range and sensitivity, the roll angle in the *x*-*z* axis and *y*-*z* axis could be estimated by the tangent angle formula from the *x*-weight, *y*-weight, and *z*-weight of the gravity, respectively. Since the sum of the acceleration must be larger than 1 g (due to acceleration by the carrier movement), the sliding filter was used here. Hence, the excessive drift of acceleration by the carrier movement could be avoided.

The voltage signal was transformed into the corresponding—first regarding the angular velocity. Then, it estimated the angular variation based on angular velocity integration. Since the horizontal rotation angle could not be calibrated by the 3-axis accelerometer, the horizontal rotation angle required the calibration drift to reduce the deviation of integration.

Since the accuracy of the angle of inclination could be higher by the 3-axis accelerometer in a static state and the accuracy of the angle of inclination could be higher by the gyro sensor in a mobile state, the Kalman filter was proposed to combine the 3-axis accelerometer with a gyro sensor to increase the accuracy of the angle of inclination in both static and mobile states. By using the self-adapting Kalman filter, the angular variation of the carrier could be calculated by adjusting the ratio of inclination angle to the ration angle.

After the sensing data were processed by the Kalman filter, the swing trend of the elbow could be observed by a graph. Since the upper and lower arms may swing together while measuring the swing of the elbow, the signals in both the upper arm and forearm need to be calibrated simultaneously. However, the offset of the fiducial value could occur, since measuring the device could not be fixed in the same location and angle until each round of measurement is completed. Hence, the overall signals needed to be calibrated based on the fiducial value to show the correct swing signal of the elbow.

The accelerometer signal could be transformed into an angle by the *x*-axis, *y*-axis, and *z*-axis weights of gravity. While the object was flattened on the table, the object was rotated based on the *y*-axis. While the *x*-axis and *y*-axis of the object were horizontal to the plane, and the *z*-axis was vertical to the plane, it fell vertically downward with 1 g of gravity on the *z*-axis. While the object was tilted or rotated, the downward gravity was balanced on the *x*-axis, *y*-axis, and *z*-axis, respectively. The acceleration values of the object in the *x*-axis (*a_x_*), *y*-axis (*a_y_*), and *z*-axis (*a_z_*) could be calculated based on (1). After the acceleration values were obtained, the angle of inclination of the object (φ) could be calculated (2).

(1) $$1g=\sqrt{a_{x}^{2}+a_{y}^{2}+a_{z}^{2}}$$

(2) $$\varphi =\tan^{-1}\frac{a_{x}}{a_{z}}\times \frac{180}{\pi }$$

The accelerometer may vibrate and the position of the accelerometer may be horizontal, with little slope, in different environments. The weights of gravity occurred on the *x*-axis, *y*-axis, and *z*-axis, respectively. The *a_x_*, *a_y_*, and *a_z_* could not be zero while the accelerometer was stable. The *a_x_*, *a_y_*, and *a_z_* were different at each time. To eliminate the above noise in the accelerometer and improve the stability and accuracy of the signal, the signal was smoothed by a simple moving average filter (SMAF). In the SMAF, the average value was calculated from the total data in a time interval to smooth the signal. The new data were added in a first-in-first-out manner (FIFO). Although the signal could be smoothed by SMAF, the data variation could not be observed. Hence, the weighted moving average filter (WMAF) was used to observe the data variation. In WMAF, each datum had its weight. The weights of the data were higher, while the data were close to the tail of the FIFO queue.

By a gyroscope, the rotation angle of the object in the *k*th unit time could be calculated by the product of the angular velocity ($\omega_{kth}$) in the *k*th time unit and the unit time of the system loop (Δ*t*). The total rotation angle of the object ($\theta_{TTL}$) in *n* unit times was derived as (3).

(3) $$\theta_{TTL}=\sum_{kth=1}^{n}\omega_{kth}\times \Delta t$$

In the simple moving average filter (SMAF) the average value was calculated by the sum of all data in a period of time, as shown in Equation (4), where *x_SMAF_* is denoted as the angle value after the SMAF and *x_n_* is denoted as the angle at time *n*. While the new data were inserted, they were placed at the end of the data queue. Then, the data in this queue moved forward one space. Finally, the data at the front of the data queue were removed (first-in-first-out (FIFO)). Although the data curve could be smooth, it could not show the variation of data. Hence, the weighted moving average filter (WMAF) was used to solve this problem. In WMAF, the higher weight was assigned to the newer data, as shown in Equation (5), where $\varphi_{WMAF}$ is denoted as the angle of inclination after WMAF and $\varphi_{k}$ is denoted as the angle of inclination at time *k*.

(4) $$x_{SMAF}=\frac{x_{1}+x_{2}+\ldots +x_{n}}{n}$$

(5) $$\varphi_{WMAF}=\frac{\sum_{k=1}^{10}k\times \varphi_{k}}{\sum_{k=1}^{10}k}$$

The systems included many kinds of sensors. However, the data captured by these sensors were often uncertain. The estimated value was often much different from the actual value. Hence, the Kalman filter was developed to address these issues. In the Kalman filter, three phases needed to be processed. The first phase was estimation, the second phase was measurement, and the final phase was update. In the estimation phase, the value of φ was calculated by the dynamic model of the system, as shown in Equation (6). At the same time, the Kalman gain (*KG*) was calculated by the estimate uncertainty (*EU*) and measurement uncertainty (*MU*), as shown in Equation (7). The $\psi_{t_{0},t_{1}}$ is denoted as the predicted angle from the state of *t*_0_ to the state of *t*_1_. The $w_{t_{0}}$ is denoted as the angle speed at *t*_0_. $KG_{t_{1}}$ is denoted as *KG* at *t*_1_. *EU*_1_ is denoted as *EU* at *t*_1_. *MU*_1_ is denoted as *MU* at *t*_1_. The initial value of the *EU* was determined by sensors or users manually. After several iterations of the Kalman filter, the optimal value of the *EU* could be obtained. *MU* was the variance of the measurement value. In the measurement phase, the optimal predicted value could be calculated by $\varphi_{t_{1}}$, $\psi_{t_{0},t_{1}}$, and *KG* after WMAF, as shown in Equation (8). Finally, in the update phase, the predicted *EU* was updated by *KG* and then the system status in the next round was predicted by the dynamic model of the system, as shown in Equations (9) and (10), where $\varphi_{t_{1}}$ is denoted as the angle of inclination after WMAF at *t*_1_.

(6) $$\psi_{t_{0},t_{1}}=\omega_{t_{0}}\times \Delta t$$

(7) $$KG_{t_{1}}=\frac{EU_{t_{1}}}{EU_{t_{1}}+MU_{t_{1}}}$$

(8) $$\psi_{t_{1},t_{1}}=\psi_{t_{0},t_{1}}+KG_{t_{1}}\left(\varphi_{t_{1}}-\psi_{t_{0},t_{1}}\right)$$

(9) $$EU_{t_{2}}=\left(1-KG_{t_{1}}\right)\times EU_{t_{1}}$$

(10) $$\psi_{t_{1},t_{2}}=\psi_{t_{1},t_{1}}+\omega_{t_{1}}\times \Delta t$$

Since the 6-axis sensor may be used in the non-horizontal state initially, the standardization correction of the swing angle of the joint was needed by the initial zero-setting. Since the calculation time of the initial value was about 1 s, the previous 60 records could not be used based on sampling frequency. The basic calibration value was calculated by capturing the 61st record to the 120th record between 1 and 2 s. The position of the waveform could be close to zero and the standardization correction could be completed. Hence, the waveform could be the swing waveform of the joint. The swing angle of the joint could be estimated by capturing the high peak value and low peak value of the waveform with the standardization correction.

The verified experimental results were evaluated with static sensing and the simulated arm, respectively, showing that the angle measured by the ROMSS was verified within the acceptable range. In the static sensing verification, it aimed to calculate the deviation of the measured angle between the ROMSS and the commercial standard digital goniometer. In the simulated arm sensing verification, it aimed to calculate the motion angle in the fixed simulated arm to verify the stability of the dynamic measurement.

**Figure 3:**
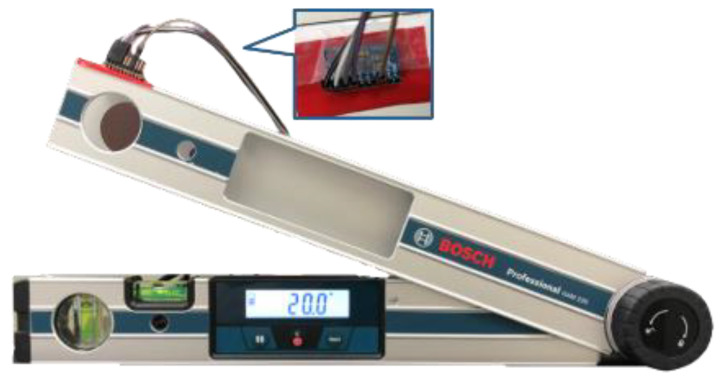
ROMSS verification in static sensing.

In the static sensing verification, the commercial standard digital goniometer, BOSCH GAM 220 (BOSCH, Nairobi, Kenya), was used. Initially, the digital goniometer was placed on the desktop, horizontally. The sensor was pasted in the digital goniometer, as shown in Figure 3.

**Figure 4:**
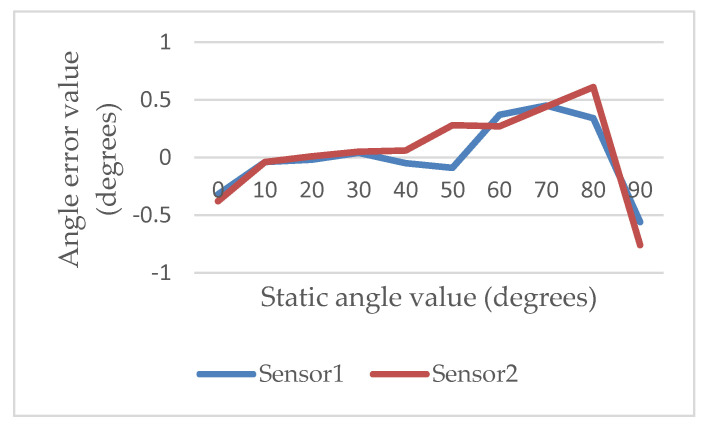
Deviation from 0 to 90 degrees in the ROMSS.

**Figure 5:**
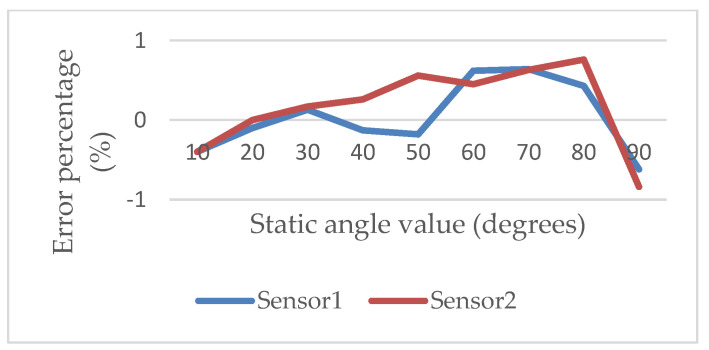
Percent deviation from 0 to 90 degrees in the ROMSS.

Then, the digital goniometer was moved to the fixed angle from 0 to 90 degrees with two inertial sensors to verify the deviation of the measured angle. In each verification round, a predefined angle was measured 3 times. The interval between the two predefined angles was 10 degrees. Each measured time was 30 s. The sampling frequency was 60 Hz. The intermittent time in each round was 10 s. The experimental results showed that the maximal deviation was −0.76 degrees while the angle of inclination of the second inertial sensor was 90 degrees, as shown in Figure 4. The average percent deviation was −0.84%, as shown in Figure 5. It was proven that the angle measured by our ROMSS was reliable in static sensing.

Based on StatCounter [27], Android and iOS were the two most popular mobile device operating systems in the world from March 2020 to March 2021. The market share of Android and iOS was 71.83% and 27.43%, respectively. Since the market share of Android was more than twice the market share of iOS, Android was used in this paper.

In the ROMSS, the microcontroller was used—Bluno Nano with Android Studio 4.12. The integrated development environment firmware of the ROMSS was divided into three parts—integrated development environment toolbar, program editing, and compilation message display. In the integrated development environment toolbar, the tools button included files and project adding, virtual machine manager, and SDK. The data could be uploaded to the mobile phone or a virtual machine by clicking the right green arrow button. The C/C++ code program was written, compiled, and uploaded to Bluno Nano in the program editing. The error and alarm messages were shown in the compilation message display. A print message could be added to the code to show a single variable while the program was running. The data could be shown by the app in real-time and uploaded to the SQLite database while the measurement was running. The users could view the data through the DB browser applications. The data were transferred to an Excel file to automatically upload to Firebase. The medical staff could easily track the rehabilitation statuses of the patients. Our system could limit the range of movement of the simulated arm by using metal clips based on the actual ROM of the human body and references.

**Figure 6:**
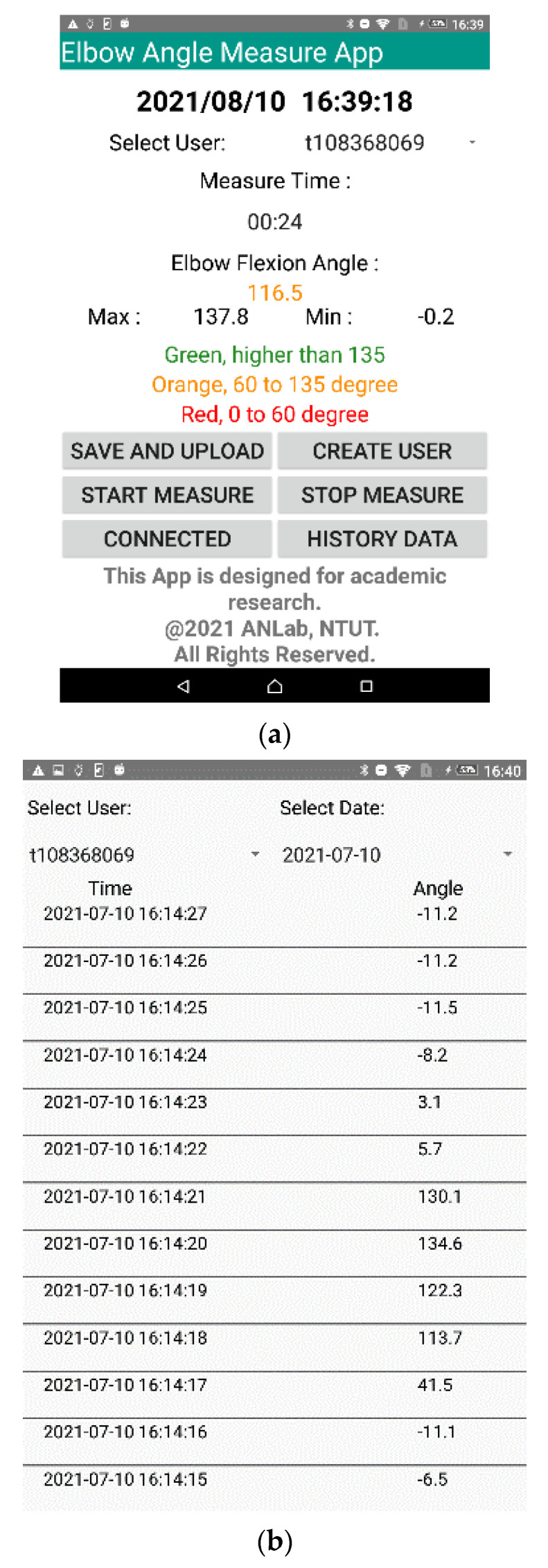

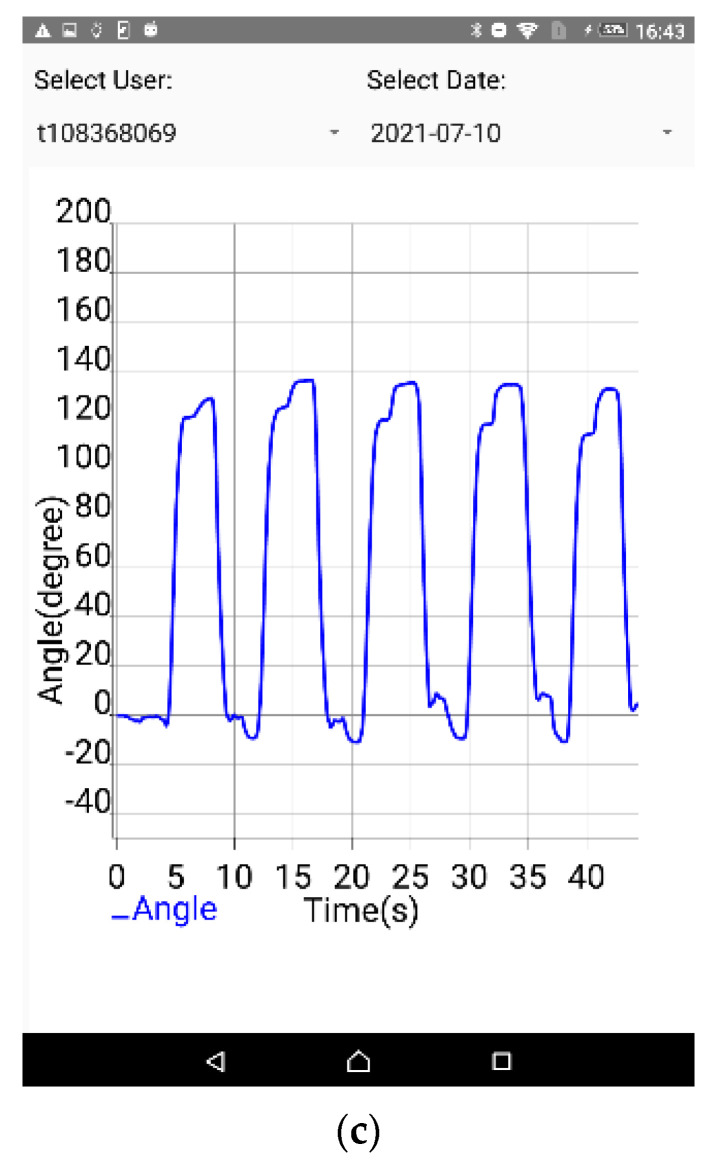
User interface of the ROMSS app. (**a**) Display current rehabilitation information, upload rehabilitation data, and browse historical records. (**b**) History query in the column. (**c**) History query in waveform.

**Figure 7:**
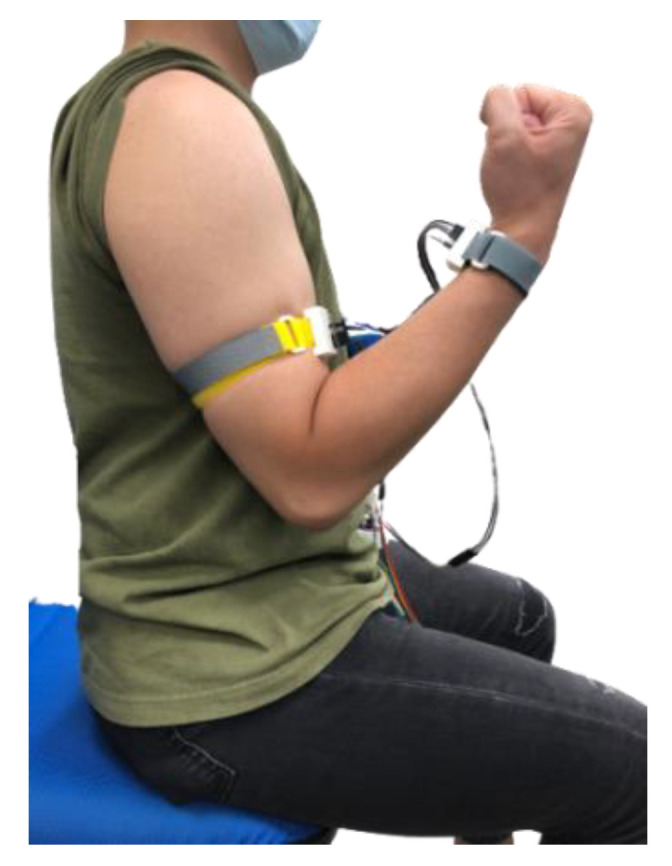
Measurement scenarios of the ROMSS.

In the ROMSS app, the data could immediately be displayed on a mobile phone as well as stored in the SQLite database of a mobile phone (to be tracked by users). The historical records could be displayed with data visualization (to be readable, such as column descriptions and waveform, as shown in Figure 6b,c). Moreover, the historical records could be displayed by the DB browser, as shown in Figure 6a. In the ROMSS app, the Excel file could be automatically uploaded to Firebase when clicking the upload button. Hence, the medical staff could inquire about the historical records of patients and track the rehabilitation statuses of patients remotely, as shown in Figure 7. The data were transferred to an Excel file to automatically upload to Firebase. Thus, the medical staff could easily track the rehabilitation statuses of patients.

**Figure 8:**
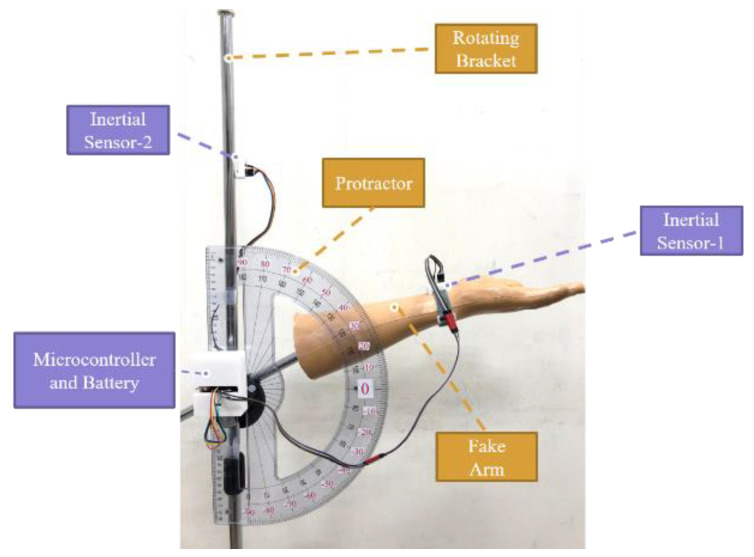
ROMSS verification in the simulated arm.

Since the elbow is the center point for the buckling and stretch of the arm, the angle was measured in a simulated arm by a swivel bracket to evaluate the accuracy of the dynamic measured angle, as shown in Figure 8. In Figure 8, the educational fake arm was simulated as a human forearm. The rotation point of the swivel bracket was simulated as the human elbow. The swivel bracket was simulated as a human upper arm. The anticlockwise rotation of the simulated arm defined the buckling as a positive value. The clockwise rotation of the simulated arm defined the stretch as a negative value. The motion range of the simulated arm was constrained by the C-type metal clip. Since the maximal rotation range of the simulated arm was smaller than the maximal angle of the C-type metal clip due to a smaller simulated arm, the deviation of the measured angle in the simulated arm was deleted. Our system could limit the range of movement of the simulated arm by using metal clips based on the actual ROM of the human body and references.

In each measurement, the simulated arm was buckled from the initial angle to the predefined angle under 3 s and then returned to the initial angle under 3 s. There were five measurements in the same predefined angle in each round. The interval of the measured angle was 10 degrees (from 10 to 60 degrees), respectively. Each measured time was 10 s. The sampling frequency was 10 Hz. The intermittent time in each round was 5 s.

In this paper, the angle of the simulated arm was measured by the ROMSS and the standard goniometer under 10 times (test cycle TC1-TC10). The measurements ranged from 0 to 140 degrees by stretch and buckling [13]. After recording on a camera, the value of the RIMSS could be displayed digitally (via playback). To examine the validity and reliability, the measured angle was checked by a standard goniometer per 10 degrees. The measured method is shown in Figure 4. The measured parameters are listed in Table 1.

## 3. Results and Discussion

The experimental results are listed in Table 2, showing that the maximal standard deviation was ±0.87 degrees and the angle of stretch was 30 degrees. The maximal mean error was 0.58 degrees and the angle of stretch was 50 degrees. The maximal standard deviation and maximal mean error were below 1 degree.

The current research related to the standard goniometer almost focused on empirical validity. The empirical validity was calculated by comparing the proposed measurement to the golden rule. Radiography was used by the optimal golden rule to measure the joint range of motion, such as Pearson’s product-moment correlation coefficient. Pearson’s product-moment correlation coefficient is defined as *r* in this paper. While the value of *r* was higher, the authenticity of measurement for the joint range of motion was higher. In [11,14], it was proven that the measured results were valid by comparing the standard goniometer to radiography. Hence, the *r* by our proposal was compared to the measured value by a standard goniometer to ensure the consistency of measured results.

To ensure the consistency of values between two different continuous measurements, reliability was used in this paper. While the elbow range of motion (measured by the same measured method) was almost the same in each round, the reliability of the measured results was high. The reliability of measured results could be denoted as the standard deviation. While the standard deviation was lower, the statistical dispersion and measurement error were lower. The standard deviation was also subsequently discussed.

**Figure 9:**
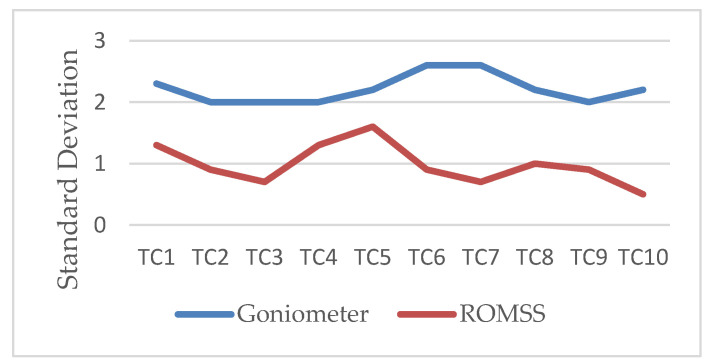
Standard deviation under the goniometer and the ROMSS in the simulated arm (test cycle 1–10).

In [28], the authors showed that the validity was good while the value of *r* was from 0.90 to 0.99. In this paper, the value of *r* was 0.90. Hence, it was proven that the correlation between our ROMSS and the standard goniometer was good. The standard deviation of the standard goniometer was from 2 to 2.6 degrees and the standard deviation of the ROMSS was from 0.5 to 1.6 degrees, as shown in Figure 9, showing that the validity of the ROMSS was higher than the validity of the standard goniometer.

In this paper, the proposed ROMSS avoided the standard deviation by manual measurements and could be set up and measured easily without hands. The ROMSS instrument was below 120 g and lighter than the electronic goniometer. The accuracy and reliability in the ROMSS were almost the same as in the electronic goniometer. The ROMSS modules were mature products on the market. The ROMSS modules, compared to electronic textiles and fiber sensing, were stable and easy to obtain. The hardware cost of the ROMSS was below USD 70. The standard deviations in image recognition, motion capture, and the Google Play app were all over 10 degrees. Since the standard deviation in the ROMSS was less than 1 degree, the ROMSS we proposed was more accurate. Although the angles measured by the mobile phone and Microsoft Kinect sensor were simple, the volumes were often large and the weights were often heavy. Moreover, they were not prone to be built. The average standard deviations in the mobile phone and Microsoft Kinect sensor were over 10 degrees. The angles measured by image recognition with a photograph and video were prone to personal errors. Hence, the ROMSS was proposed in this paper. In the ROMSS, the measured data could automatically be uploaded to a cloud database in real-time. Patients could query the measured data at home by the designed app. Medical staff could track the status of rehabilitation by an app remotely to revise the rehabilitation for each patient in real-time.

## 4. Conclusions

To avoid measurement deviations from human error when using a standard goniometer, a range of motion sensing system (ROMSS) was developed by composing of an inertial sensor and Bluno Nano-embedded board. In the ROMSS, a Kalman filter was integrated with a gyroscope and accelerometer to calculate the optimal angle. The sensing data could be uploaded to SQLite on a mobile device via Bluetooth. The users could query their rehabilitation data in real-time via the app we designed. The sensing data could also be uploaded to a cloud database via the internet (to be queried by medical staff, remotely).

By pasting our two inertial sensors on the commercial digital goniometer, the maximal average deviation between the inertial sensors and digital goniometer was −0.76 degrees while the digital goniometer moved to the predefined measured angle. In the same condition, the maximal percent deviation was −0.85%. It showed that the average deviation and percent deviation were less than ±1%. To evaluate the accuracy of the dynamic measured angle, the ROMSS device was fixed in the simulated arm. We evaluated the dynamic deviation by turning the simulated arm in the fixed range of the angle back and forth. It showed that the maximal average deviation was 0.58 degrees and the maximal percent deviation was ±0.87%. Since Pearson’s product-moment correlation coefficient (*r*) between the ROMSS and the digital goniometer was 0.90, the validity of the ROMSS was good. In the experimental results, the standard deviation of the digital goniometer was between ±2 and ±2.6 degrees. the standard deviation of the ROMSS was between ±0.5 and ±1.6 degrees. It was proven that the reliability of the ROMSS was higher than *that* of the digital goniometer.

To achieve the goal of the ROMSS with lower hardware costs, the sensors and embedded board were used on the market. Hence, the function and performance of the sensors and embedded board were restricted. Moreover, the volume and electronic pin could not be used for the ROMSS directly. Hence, the shell mechanism of ROMSS needed to be implemented by a 3D printer and fixed on the body with straps. Since the sensor could not be pasted close to the skin, measurement errors must have occurred. In addition, the shell mechanism of the lithium battery needed to be implemented by a 3D printer since the volume of the lithium battery in the embedded board was large. Therefore, the ROMSS device was complete and reliable once the sensors were developed by the film type and integrated with an embedded board with lithium battery. In the current ROMSS device, two inertial sensors were used. Once the number of inertial sensors increased, the accuracy of the measured angle increased. Moreover, it could be applied to human posture recognition and gait recognition.

In the future, the sensor, battery fixing, battery charging, battery lifespan, data collection, and presentation will continuously improve via discussions with medical staff during the development process.

IRB will be applied to examine the difference between rehabilitation with the ROMSS and rehabilitation without the ROMSS for patients with elbow fractures. We will also examine patient satisfaction regarding the functions in our app, such as real-time data display, and uploading. Moreover, the angle under the pronation and supination of the arm will be measured since the angle was only measured under the stretch and buckling of the arm in this paper. Finally, IRB will be applied for rehabilitation regarding the lower limbs, neck, frozen shoulders, hemophilia, and cancer, respectively.

## Figures and Tables

**Table 1:** Measured parameters in the ROMSS with the simulated arm.

Name	Value (Unit)
Number of measurements	10 times
Number of motions	10 (round/time)
Frequency of motion	1 (second/round)
Sampling frequency	10 Hz
Intermittent time	10 (second/time)

**Table 2:** Dynamic standard deviation and mean error in the ROMSS with the simulated arm.

Measured Angle (*MA*)	Average Angle with Standard Deviation (*AA*)		Average Standard Deviation (ACD = MA − AA)	
	Buckling	Stretch	Buckling	Stretch
±10	10.28 ± 0.41	−9.67 ± 0.76	0.28	0.33
±20	20.33 ± 0.11	−20.29 ± 0.38	0.33	−0.29
±30	30.39 ± 0.35	−29.88 ± 0.87	0.39	0.12
±40	39.92 ± 0.20	−40.08 ± 0.18	−0.08	−0.08
±50	50.15 ± 0.54	−49.42 ± 0.01	0.15	0.58
±60	60.12 ± 0.18	−59.55 ± 0.82	0.12	0.45

## Data Availability

Not applicable.
